# Supplementary material for: Predictors of health-related quality of Life for COVID-19 survivors living in Dhaka, Bangladesh: A repeated Follow-Up after 18 months of their recovery
Source: PLOS Glob Public Health. 2024 Aug 28;4(8):e0003472. doi: 10.1371/journal.pgph.0003472 (PMC11356435; doi:10.1371/journal.pgph.0003472)
Supplement: S3 Fig — (DOCX) [file pgph.0003472.s003.docx]

**
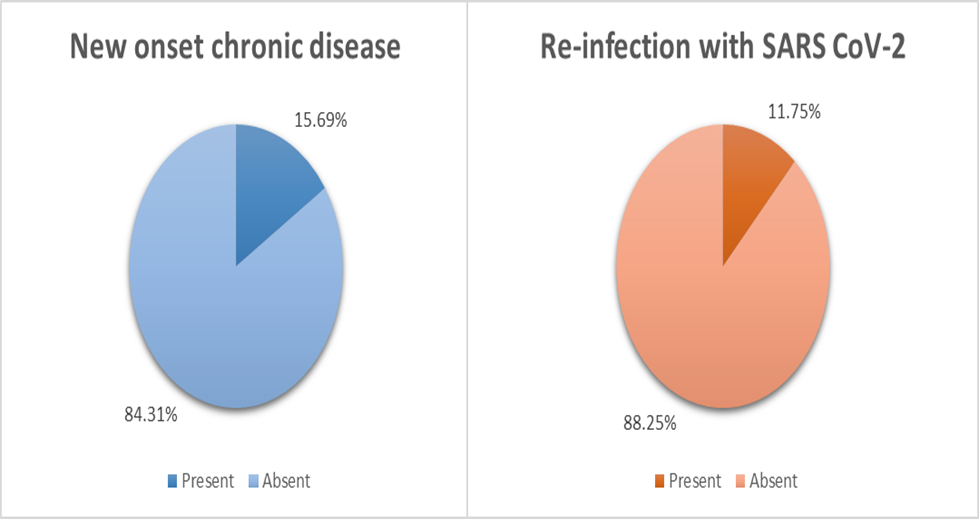
**

**S3 Fig: Onset of new chronic disease and percentage of re-infection among the recovered COVID-19 participants during second follow-up**
