# Supplementary material for: Predictors of health-related quality of Life for COVID-19 survivors living in Dhaka, Bangladesh: A repeated Follow-Up after 18 months of their recovery
Source: PLOS Glob Public Health. 2024 Aug 28;4(8):e0003472. doi: 10.1371/journal.pgph.0003472 (PMC11356435; doi:10.1371/journal.pgph.0003472)
Supplement: S1 Table — (DOCX) [file pgph.0003472.s004.docx]

**S1 Table: Comparison of quality of life between baseline and follow-up interviews in relation to presence or absence of individual chronic diseases**

| **Variables** | | **Physical** | | | **Psychological** | | | **Social** | | | **Environmental** | | |
| --- | --- | --- | --- | --- | --- | --- | --- | --- | --- | --- | --- | --- | --- |
|  |  | **1^st^ visit** | **2^nd^ visit** | **p value** | **1^st^ visit** | **2^nd^ visit** | **p value** | **1^st^ visit** | **2^nd^ visit** | **p value** | **1^st^ visit** | **2^nd^ visit** | **p value** |
|  |  | **Mean (SD)** | **Mean (SD)** |  | **Mean (SD)** | **Mean (SD)** |  | **Mean (SD)** | **Mean (SD)** |  | **Mean (SD)** | **Mean (SD)** |  |
| **Hypertension** | No | 69.61 (13.94) | 64.18 (13.17) | **<0.001** | 64.08 (14.96) | 64.72 (14.10) | 0.27 | 64.07 (19.67) | 64.15 (17.82) | 0.91 | 63.91 (12.33) | 66.71 (12.01) | **<0.001** |
|  | Yes | 61.30 (13.25) | 59.36 (13.42) | 0.06 | 56.39 (16.26) | 60.13 (13.42) | **0.003** | 62.41 (18.43) | 64.18 (15.78) | 0.19 | 61.31 (13.14) | 65.43 (12.62) | **<0.001** |
|  | p-value | **<0.001** | **<0.001** |  | **<0.001** | **<0.001** |  | 0.23 | 0.98 |  | **0.003** | 0.133 |  |
| **Diabetes Mellitus** | No | 69.78 (13.77) | 64.25 (13.42) | **<0.001** | 64.19 (15.09) | 64.64 (13.99) | 0.44 | 64.41 (19.75) | 64.57 (17.69) | 0.82 | 63.59 (12.25) | 66.82 (12.22) | **<0.001** |
|  | Yes | 60.32 (13.36) | 58.93 (12.27) | 0.16 | 55.68 (15.51) | 60.30 (13.56) | **<0.001** | 61.01 (17.82) | 62.52 (16.17) | 0.27 | 62.52 (13.62) | 64.96 (11.75) | **0.04** |
|  | p-value | **<0.001** | **<0.001** |  | **<0.001** | **<0.001** |  | **0.014** | 0.099 |  | 0.233 | **0.032** |  |
| **Heart Diseases** | No | 68.93 (14.10) | 63.48 (13.43) | **<0.001** | 63.20 (15.39) | 64.07 (14.08) | 0.12 | 64.46 (19.61) | 64.10 (17.67) | 0.58 | 63.71 (12.25) | 66.84 (12.12) | **<0.001** |
|  | Yes | 58.22 (11.18) | 60.33 (12.47) | 0.12 | 55.80 (15.48) | 60.92 (13.03) | **<0.001** | 56.93 (16.13) | 64.63 (14.79) | **<0.001** | 60.26 (14.62) | 62.88 (11.85) | 0.13 |
|  | p-value | **<0.001** | **0.014** |  | **<0.001** | **0.019** |  | **<0.001** | 0.755 |  | **0.004** | **<0.001** |  |
| **Asthma** | No | 69.45 (14.07) | 63.92 (13.32) | **<0.001** | 64.10 (15.11) | 64.17 (14.11) | 0.90 | 65.15 (19.20) | 64.33 (17.41) | 0.21 | 63.80 (12.11) | 66.94 (11.98) | **<0.001** |
|  | Yes | 59.62 (11.82) | 59.25 (12.96) | 0.76 | 53.97 (15.08) | 61.63 (13.31) | **<0.001** | 56.24 (18.86) | 63.25 (17.39) | **<0.001** | 61.14 (14.40) | 63.83 (12.68) | 0.07 |
|  | p-value | **<0.001** | **<0.001** |  | **<0.001** | **0.020** |  | **<0.001** | 0.430 |  | **<0.001** | **<0.001** |  |
| **Chronic Kidney Disease (CKD)** | No | 68.27 (14.30) | 63.37 (13.32) | **<0.001** | 62.82 (15.35) | 63.94 (14.00) | **0.04** | 64.12 (19.38) | 64.34 (17.42) | 0.73 | 63.71 (12.48) | 66.36 (11.96) | **<0.001** |
|  | Yes | 60.86 (10.13) | 59.70 (13.73) | 0.55 | 56.35 (17.81) | 60.65 (13.95) | 0.14 | 56.71 (18.83) | 60.92 (16.95) | 0.17 | 57.56 (12.27) | 67.98 (15.04) | **<0.001** |
|  | p-value | **<0.001** | **0.03** |  | **0.001** | 0.064 |  | **0.003** | 0.121 |  | **<0.001** | 0.290 |  |
| **Cancer** | No | 68.33 (14.28) | 63.42 (13.17) | **<0.001** | 62.98 (15.41) | 64.16 (13.77) | **0.03** | 64.20 (19.43) | 64.38 (17.33) | 0.77 | 63.40 (12.20) | 66.56 (12.03) | **<0.001** |
|  | Yes | 58.79 (8.33) | 63.17 (13.37) | 0.84 | 52.29 (14.92) | 55.86 (16.40) | 0.21 | 54.24 (16.76) | 59.62 (18.43) | 0.10 | 62.78 (18.09) | 64.10 (14.10) | 0.69 |
|  | p-value | **<0.001** | **0.004** |  | **<0.001** | **<0.001** |  | **<0.001** | 0.042 |  | 0.711 | 0.133 |  |
