# Supplementary material for: Predictors of health-related quality of Life for COVID-19 survivors living in Dhaka, Bangladesh: A repeated Follow-Up after 18 months of their recovery
Source: PLOS Glob Public Health. 2024 Aug 28;4(8):e0003472. doi: 10.1371/journal.pgph.0003472 (PMC11356435; doi:10.1371/journal.pgph.0003472)
Supplement: S1 Questionnaire — (PDF) [file pgph.0003472.s005.pdf]

Quality of Life assessment of patients with COVID-19 after recovery- A nationwide study in Bangladesh

**Identifying information**		**Date of interview:____/____/ 2020**		
1.	ID: [ ][ ][ ][ ][ ][ ]			
2.	Name of the Respondent:			
3.	Address (Present): District-	Division-		
**Demographic information**				
	**Variable/Question/Statement**		**Skip**	**Code**
4.	Age (in completed years):__________Years			
5.	What is your gender:	1. Male<br>2. Female		
6.	What is your residence?	1. Rural<br>2. Urban<br>3. Semi-urban		
7.	What is the highest education you received?	6. No formal education<br>7. Primary<br>8. Up to SSC<br>9. Up to HSC<br>10. Graduation<br>11. Post-graduation		
8.	What is your current occupation?	1. Service<br>2. Business<br>3. Farmer<br>4. Housewife<br>5. Student<br>6. Unemployed<br>7. Others. Please specify………..		
9.	Are you a healthcare worker?	1. Yes<br>2. No		
10.	What is your marital status	1. Single<br>2. Married<br>3. Separated<br>4. Divorced<br>5. Widowed/widower		

11.	Monthly family (household) income (BDT)	………………..		
**Personal history, comorbidities and symptom profile**				
12.	Were you admitted to hospital due to COVID?	1. Yes<br>2. No		
13.	Do you smoke?	1. Yes<br>2. No<br>3. Past smoker		
14.	Do you have any chronic diseases?	1. HTN -Yes / No<br>2. Diabetes -Yes / No<br>3. Heart disease (IHD/VHD/Others) -Yes / No<br>4. Asthma/COPD -Yes / No<br>5. CKD -Yes / No<br>6. Cancer -Yes / No		
15.	Has anyone in your family been hospitalized to the ICU due to covid-19 infection during the corona pandemic?	1. Yes<br>2. No.		
16.	Has any of your family member died while undergoing treatment at home or in ICU due to corona?	1. Yes<br>2. No		
17.	Have you been infected with Covid again after being affected previously?	1. Yes<br>2. No		
18.	Once you are infected with Cavid-19, do you worry to be infected again?	1. Yes<br>2. No		
**COVID19 Vaccination:**				
1.	Did you take vaccine?	1. Yes<br>2. No		
If yes, how many doses have you taken?		1. 1 dose<br>2. 2 doses		

2.	When did you take the first dose of vaccine?	1.Within 6 months<br>2. Before 6 months		
3.	Which company's vaccine did you take as your first dose?	1.Oxford–AstraZeneca<br>2.Sinopharm<br>3.Moderna<br>4.Pfizer		
4.	When did you take the second dose of vaccine?	1.Within 6 months<br>2. Before 6 months		
5.	Which company's vaccine did you take as your second dose?	1.Oxford–AstraZeneca<br>2.Sinopharm<br>3.Moderna<br>4.Pfizer		
**Some Other Relevant Questions:**				
1.	Have you been infected with covid after getting vaccinated?	1. Yes, if yes, after which dose? First dose or second dose?<br>2. No		

WHO BREF Quality of Life Questionnaire

Instructions

This assessment asks how you feel about your quality of life, health or other areas of your life after you have been infected with covid-19. Please answer all the questions. If you are unsure about which response to give to a question, please choose the one that appears most appropriate. This can often be your first response. If you feel difficulty understanding the questions, we will help you regarding that.

(Explain it to the respondent: You should circle the number that best fits how much support you've got from others over the last two weeks. You have to choose a number between 1 to 5 ranging from very poor=1, poor=2, neither poor nor good=3, good=4 and very good=5.)

Please read each question, assess your feelings, and circle the number on the scale for each question that gives the best answer for the participants

		Very poor	Poor	Neither poor nor	Good	Very good
				good		
1	How would you rate your quality of life?	1	2	3	4	5
		Very dissatisfied	Dissatisfied	Neither satisfied nor dissatisfied	Satisfied	Very satisfied
2	How satisfied are you with your health?	1	2	3	4	5
		Not at all	A little	A moderate amount	Very much	An extreme amount

The following questions will ask you to know your experience in the last few days after you have been infected with covid-19.

3	To what extent do you feel that physical pain prevents you from doing what you need to do?	1	2	3	4	5
4	How much do you need any medical treatment to function in your daily life?	1	2	3	4	5
5	How much do you enjoy life?	1	2	3	4	5
6	To what extent do you feel your life to be meaningful?	1	2	3	4	5
7	How well are you able to concentrate?	1	2	3	4	5
8	How safe do you feel in your daily life?	1	2	3	4	5
9	How healthy is your physical environment?	1	2	3	4	5

The following questions ask about how completely you experience or were able to do certain things in the last two weeks after being diagnosed with covid.

		Not at all	A little	Moderately	Mostly	Completely
10	Do you have enough energy for everyday life?	1	2	3	4	5
11	Are you able to accept your bodily appearance?	1	2	3	4	5
12	Have you enough money to meet your needs?	1	2	3	4	5
13	How available to you is the information that you need in your day-to-day life?	1	2	3	4	5
14	To what extent do you have the opportunity for leisure activities?	1	2	3	4	5
		Very poor	Poor	Neither Good Not Poor	Good	Very good
15	How well are you able to get around?	1	2	3	4	5

The following questions ask you to say how good or satisfied you have felt about various aspects of your life over the last two weeks.

		Very dissatisfied	Dissatisfied	Neither satisfied nor dissatisfied	Satisfied	Very satisfied
16	How satisfied are you with your sleep?	1	2	3	4	5
17	How satisfied are you with your ability to perform your daily living activities?	1	2	3	4	5
18	How satisfied are you with your capacity for work?	1	2	3	4	5
19	How satisfied are you with yourself?	1	2	3	4	5
20	How satisfied are you with your personal relationships?	1	2	3	4	5
21	How satisfied are you with your sex life?	1	2	3	4	5
22	How satisfied are you with the support you get from your friends?	1	2	3	4	5
23	How satisfied are you with the conditions of your living place?	1	2	3	4	5
24	How satisfied are you with your access to health services?	1	2	3	4	5
25	How satisfied are you with your transport?	1	2	3	4	5

The following question refers to how often you have felt or experienced certain things in the last two weeks after being infected with covid.

		Never	Seldom	Quite often	Very often	Always
26	How often do you have negative feelings such as blue mood, despair, anxiety, depression?	1	2	3	4	5

Do you have any comments about the assessment?

..
..
....

THANK YOU FOR YOUR HELP
