# Supplementary material for: Predictors of health-related quality of Life for COVID-19 survivors living in Dhaka, Bangladesh: A repeated Follow-Up after 18 months of their recovery
Source: PLOS Glob Public Health. 2024 Aug 28;4(8):e0003472. doi: 10.1371/journal.pgph.0003472 (PMC11356435; doi:10.1371/journal.pgph.0003472)
Supplement: Attachment — Submitted filename: 1. PGPH_Response to Reviewers.docx [file pgph.0003472.s008.docx]

Dear Editor,

Thank you for taking the time to review our manuscript and for providing us with detailed feedback. We are grateful to the reviewers for their thorough evaluations and insightful comments. We have carefully addressed each comment, making the necessary modifications to the manuscript accordingly. We have provided a point-by-point response directly beneath each comment to demonstrate how we have addressed the concerns raised.

**Journal Requirements**

1. Please include a complete copy of PLOS’ questionnaire on inclusivity in global research in your revised manuscript. Our policy for research in this area aims to improve transparency in the reporting of research performed outside of researchers’ own country or community. The policy applies to researchers who have travelled to a different country to conduct research, research with Indigenous populations or their lands, and research on cultural artefacts. The questionnaire can also be requested at the journal’s discretion for any other submissions, even if these conditions are not met. Please find more information on the policy and a link …………. Please upload a completed version of your questionnaire as Supporting Information when you resubmit your manuscript.

**Response:** Thank you. PLOS’s questionnaire on inclusivity in global research and a completed version of the questionnaire have been attached as supplementary.

2. In the ethics statement in the Methods, you have specified that verbal consent was obtained. Please provide additional details regarding how this consent was documented and witnessed, and state whether this was approved by the IRB.

**Response:** Thank you for the comment. Since this was a telephone interview-based study, we obtained verbal consent from the participants. Data collectors gave the participants a comprehensive briefing about the project over the phone. The interview only proceeded after obtaining consent; otherwise, the call was terminated. Moreover, the Institutional Review Board of North South University, Dhaka, Bangladesh, approved this project. Please check lines **430-434** for detailed information.

3. 3. Please provide separate figure files in .tif or .eps format only and remove any figures embedded in your manuscript file. Please also ensure all files are under our size limit of 10MB.

**Response:** All our figures have been changed in .tif format and within 10MB.

**Reviewers’ Comments**

**Reviewer #1: Comments to Manuscript No: PGPH-D-23-02307**

First of all, thank you for giving me this opportunity. The item is acceptable. However, I leave it up to you to evaluate the conformity of the work with the magazine's guidelines. The article is acceptable given the great interest of the topic for the scientific community. The article touches on a very delicate and complex topic. However, the authors clearly described the results and discussion.

**Response:** Thank you for your valuable comments. Also, thank you so much for appreciating our work.

**Reviewer #2: Comments to Manuscript No: PGPH-D-23-02307**

Dear Authors, thank you for giving me the opportunity to read your study. In a large cohort of COVID-19 patients, you found some risk factors for worse QOL or some protective factors for better QOL. The study in interesting, and Authors should be commended for the effort. I have some suggestions that I hope they will improve the quality of your paper.

1. I recommend English proofreading because some parts are difficult to read and understand.

**Response:** Thank you for the comment. We have proofread the entire manuscript for English modification where necessary. Later, we used an advanced proofreading tool to double-check any typos and clarity-related issues and update our revised manuscript accordingly.

2. Introduction is very/too long. Please, after a general overview of the problem, focus on what you will investigate in your study.

**Response:** Thank you for your observations. We have revised our introduction section according to your suggestions. Kindly check lines **85–134**.

3. One factor that could highly impair QOL after COVID infection is the admission to the ICU. In this regard, if you feel appropriate, consider this article: J Clin Med. 2023 Jan 29;12(3):1058. DOI: 10.3390/jcm12031058. PMID: 36769705; PMCID: PMC9918008.

**Response:** Thank you for the insightful opinion. We considered and incorporated the suggested article (Reference No 31).

4. As you correctly pointed out, one of the main problems during pandemic was the limited resources especially in low income countries. You find interesting insights in this nice and appropriate in my opinion article: Acta Biomed. 2021 May 12;92(2):e2021097. DOI: 10.23750/abm.v92i2.11159. PMID: 33988143; PMCID: PMC8182622.

**Response:** Thank you for the valuable suggestion. We have incorporated this mentioned article in terms of your perceptive viewpoints (Reference No 51).

5. In discussion, please, try to reformulate this section giving more emphasis to your results. In other words, it is right to sum up literature evidence, but is also important to highlight your results.

**Response:** We revised our discussion section by emphasizing our study findings according to your suggestions. We have underscored every significant predictor in our revised manuscript. Kindly check lines **291–295, 301–304, 329–338.**
